# Brigham Fracture Intervention Team Initiatives for Hospital Patients with Hip Fractures: A Paradigm Shift

**DOI:** 10.1155/2010/590751

**Published:** 2009-08-26

**Authors:** Julie Glowacki, Mitchel B. Harris, Josef Simon, John Wright, Nikheel S. Kolatkar, Thomas S. Thornhill, Meryl S. LeBoff

**Affiliations:** Departments of Orthopedic Surgery and Medicine, Brigham and Women's Hospital, 75 Francis Street, Boston, MA 02115, USA

## Abstract

We designed, implemented, and revised the Brigham Fracture Intervention Team (B-FIT) initiatives to improve in-hospital care of fracture (Fx) patients. Effectiveness was evaluated for 181 medical records of 4 cohorts in four successive years of consecutive patients who were admitted with a fragility hip Fx. The Discharge Initiative (DI) (computer-based) includes 1200 mg calcium and 1000 IU vitamin D_3_ daily. The Admission Initiative (AI) was introduced one year later with reminders for serum 25OHD measurement, initiation of daily calcium (1200 mg) and vitamin D (800 IU), and an order for Endocrinology consultation, with an amendment for a computer-assisted reminder and a dose of D_2_ (50 000 IU). Initially, the computer-based DI was more effective (67%) than the surgeon-driven AI (33%, *P* < .001). After introduction of a computer-assisted reminder, AI effectiveness increased to 68%. The marked prevalence of vitamin D insufficiency reaffirms the importance of incorporating vitamin D recommendations in Fx care pathways.

## 1. Introduction

There is growing concern about the marked inadequacy of osteoporosis care for patients who suffer a fragility (low-energy) fracture [1]. Hip fractures increase exponentially with age and are associated with impaired mobility, loss of independence, increased hospitalizations, and mortality for up to one in four women during the year following the fracture [2]. Evidence shows that risk of fracture is increased in those who have had a first fracture [3]. A report from a Danish registry indicates that for patients with a hip fracture there was a 6-fold increase in risk of second hip fracture for women and a 9-fold increase for men [4]. An analysis of data from two longitudinal studies in the United States showed that hip fracture patients had a 2.5-fold increased risk of subsequent self-reported fragility fracture other than hip [5]. In the United States, although approximately 90% of patients with hip fractures have osteoporosis, only 19% of them receive postfracture evaluation or therapy for osteoporosis [6]. Moreover, a survey of databases of health maintenance organizations indicates that this is a widespread problem [7].

This study evaluates the effectiveness of the Brigham Fracture Intervention Team (B-FIT) initiatives, new in-hospital care initiatives designed to address osteoporosis evaluation and in-hospital management. These care pathways include a Discharge Initiative and several versions of Admissions Initiatives. Because of our earlier finding of vitamin D deficiency in patients admitted with a hip fracture [8], these initiatives include elements to identify and manage vitamin D deficiency. The goal is to attend to underlying musculoskeletal pathology that resulted in the fracture and to launch that care by the admitting orthopedic surgeons.

It is well appreciated that prolonged vitamin D deficiency in adults results in osteomalacia and hypovitaminosis D myopathy with diffuse pain, increased risk of falls and fractures, and nondisplaced pseudofractures. In addition, hyperparathyroidism secondary to hypovitaminosis D increases bone resorption and further reduces bone mineral density. These consequences of chronic vitamin D deficiency, osteomalacia, osteopenia, and osteoporosis are each associated with increased fracture risk [9–11].

It was our goal to translate those clinically relevant research findings into hospital-based initiatives to improve the care of patients admitted with a fragility fracture. A multidisciplinary team of orthopedic surgeons, endocrinologists, and investigators developed and revised Admission and Discharge Initiatives to evaluate and address the osteoporosis and vitamin D status of patients admitted with a fragility fracture of the hip. The responsible orthopedic surgeons were trained to place the orders for hospital in patients. These in-hospital care elements involved cooperation between orthopedic surgeons and endocrinologists, clinician training and reinforcement, and evolution with computer assistance. Effectiveness of these B-FIT initiatives was evaluated with audits of four annual cohorts of patients. Improvements were made in response to interim findings.

## 2. Methods

### 2.1. Study Population

With an IRB-approved protocol and its annual review, medical records of patients ≥50 years of age who were admitted with fragility fracture of the hip and proximal femur were examined. Cohorts comprised patients who were admitted during the last five months of each of 4 years in order to ensure comparable appraisals in light of interim modifications made in the Admission Pathway and potential seasonal variations. Revisions were introduced early in calendar years so that they would be well established by the time of the annual evaluation. Each patient's record was reviewed by a board-certified orthopedic surgeon to verify that the fracture represented a fragility (low energy) fracture. After review, patients with avascular necrosis (*n* = 2), high energy traumatic fracture (*n* = 3), or fracture of unspecified etiology (*n* = 2) were excluded. A total of 181 subjects were included. In the Year 2 cohort, two patients died of unrelated medical causes while in hospital and thus were not available for analysis of Discharge effectiveness. One in-hospital death occurred in the Year 4 cohort.

### 2.2. B-FIT Admission and Discharge Initiatives

The first surgeon-initiated initiative was introduced as additions to the pre-existing computer-based discharge order-set for fracture patients. The B-FIT Discharge Initiative prompts the surgeon to give written and oral instructions for daily calcium with vitamin D (600 mg elemental calcium with 200 IU vitamin D_3_, bid), a daily multivitamin (with 400 IU vitamin D_3_ and 66 mg calcium), additional daily vitamin D (400 IU vitamin D_3_), and an outpatient osteoporosis evaluation (Table 1). The total amount of supplemental vitamin D_3_ is 1200 IU daily. The total amount of supplemental calcium is 1240 mg daily.

The Admission Initiative was introduced early in year 1 in the form of a training session for orthopedic attendings and residents, distribution of laminated pocket cards, and follow-up emails to train the rotating orthopedic residents. It instructs the surgeon to order tests for serum calcium, albumin, and 25-hydroxyvitamin D [25OHD] (Nichols Advantage prior to January 1, 2005; thereafter DiaSorin Liaison), to start daily calcium carbonate with vitamin D_3_ (600 mg elemental calcium with 200 IU vitamin D_3_, bid), a daily multivitamin (with 400 IU vitamin D_3_ and 66 mg calcium), and to order an Endocrinology consultation. The total daily dose of supplemental vitamin D_3_ was 800 IU during hospitalization.

In early Year 3, amendments to the Admission Initiative were implemented. A computer-assisted reminder was deployed to appear as a pop-up screen in the fracture service database program when the details of the admission were entered and met the study criteria (fragility fracture of the hip/femur and age ≥50 years). The amendment also added the order for administration of one oral dose of 50,000 IU vitamin D_2_.

### 2.3. Measures of Effectiveness

With IRB approval and annual review, medical records were inspected for past medical history, elements of admission and discharge instructions, and details of the discharge regimen.

Effectiveness of Admission Initiative was defined as the percent of patients who had a measurement of 25OHD during hospitalization. Median values of 25OHD were calculated for each cohort. Vitamin D insufficiency was defined as <32 ng/mL 25OHD. Secondary analyses were made of percents of subjects with a record of an Endocrine consultation, of administration of 50,000 IU vitamin D_2_ while hospitalized, or with a record of past medical history of a diagnosis of osteoporosis. Effectiveness of Discharge Initiative was defined as the percent of patients who were provided with instructions for calcium/vitamin D.

### 2.4. Statistical Analysis

Statistical analysis was performed with GraphPad InStat version 3.00 for Windows 95, GraphPad Software Inc. (www.graphpad.com). Categorical data are reported as frequencies and continuous data are reported as the mean ± standard deviation, unless otherwise specified. Comparisons of frequencies were made with Fisher's Exact Test (http://www.langsrud.com/fisher.htm). All *P*-values are two-sided.

## 3. Results

Cohorts of 57, 41, 42, and 41 subjects fulfilled the study criteria (fragility fracture of the femur and older than 50 years) in the designated period in years 1, 2, 3, and 4, respectively (Table 1). These cohorts included 74 to 82% women. Review of comorbidities prior to hospitalization for fracture revealed that 15% had a pre-existing diagnosis of osteoporosis and 17% had a diagnosis of hypothyroidism. Of the subjects that had been tested for serum 25OHD concentration, 78% were found to be insufficient, having serum levels <32 ng/mL. 48% had serum 25OHD levels ≤20 ng/mL, the level commonly used to define vitamin D deficiency; 35% had serum 25OHD levels ≤15 ng/mL, a level of severe deficiency.

Effectiveness of the Admission Initiative was 33% in the months following its introduction. After deployment of a computer-assisted reminder early in year 2, there was an increase in Admission effectiveness, reaching 68% effectiveness in year 4 (*P* = .001). Additional analyses showed an increase in percent of Endocrine consultations from 7% in year 1, to 22% in year 2, to 34% in year 3, and 48% in year 4. After introduction of the element to administer a single oral dose of 50,000 IU of vitamin D_2_, 29% of the subjects received this treatment, whereas the percent was 52% in year 3 and 54% in year 4.

Effectiveness of the computer-assisted Discharge Initiative in year 1 was 67%. This was significantly greater than the 33% effectiveness of the then-newer surgeon-driven Admission Initiative (*P* < .001). In year 1, the Discharge Initiative had been in use for a year more than the Admission Initiative. In addition, the Discharge Initiative was computer prompted from its introduction. With time, there were further increases in Discharge effectiveness with statistical trends but not significance. By year 3, the Discharge Initiative had reached an effectiveness level of 83%. On the basis of having a report during hospitalization of very low 25OHD and an in-hospital consultation with an endocrinologist, many patients were discharged with a regimen that included a prescription for 50,000 IU vitamin D_2_ per week for 8 weeks, to initiate correction of the deficiency.

## 4. Discussion

This study describes our four-year experience in transforming the care of fragility fractures of the femur. Our B-FIT program development was greatly enhanced through multidisciplinary interactions and deployment of hospital-based computer-assisted enhancements of the Admission and Discharge Initiatives at Brigham and Women's Hospital. Effectiveness of these surgeon-driven orders was enhanced with computer-based tools. Recent launching of prewritten order sets is expected to further improve in-hospital management of osteoporosis. Many hospitals now have care improvement services that can expedite this.

Analysis of the Year 1 cohort revealed that the effectiveness of the Discharge Initiative (67%) was twice that of the Admission Initiative (33%, *P* < .001). This finding is attributable to the fact that the Discharge Initiative was implemented one year earlier than the Admission Initiative, and that the Discharge Initiative was integrated into the hospital computer-based discharge-order system. Greater clinician familiarity and education can contribute to the yearly increases in both pathways.

The first mechanism that was used to introduce the Admission Initiative consisted of training sessions for orthopedic attendings and residents, laminated pocket cards, and periodic email reminders. Feedback from residents indicated that it was inconvenient to manually integrate the information on the cards with the hospital's computer-based order system. This led to an enhancement of the Initiative. In early year 2, a pop-up screen displaying the Admission Initiative was developed for the orthopedic trauma service database program to be activated upon entry of the details of the admission that met the study criteria. Effectiveness increased in each subsequent cohort.

The finding of vitamin D insufficiency in this study is consistent with other investigations of patients with fragility fractures. In an evaluation of United States community-living postmenopausal women with hip fracture and no causes of secondary osteoporosis, we reported that 50% of them had extreme vitamin D deficiency (25OHD ≤ 12 ng/mL) [8]. With the current threshold of 32 ng/mL, 90% of those subjects are classified as vitamin D insufficient [12]. In a larger study of 110 women in Boston and Baltimore with fragility fracture of the hip, vitamin D insufficiency was defined by the more recent threshold (<32 ng/mL), which was present in 96% [12]. Our recent report of a series of consecutive patients with fragility fractures showed vitamin D sufficiency in only 22% [13]. A recent multinational study showed that 90% of 385 subjects with a hip fracture had serum 25OHD levels <32 ng/mL [14]. This reaffirms that vitamin D insufficiency is an international concern for hip fracture patients. A United States study of women and men with fragility fracture of the hip, humerus, spine, rib, or wrist indicated that nearly all had vitamin D insufficiency [15]. Likewise, analysis of data from NHANES III showed that serum 25OHD was significantly related to reduced hip fractures in non-Hispanic white adults >65 year of age [16]. Data associating lower vitamin D levels with reduced muscle strength and increased risk of falls [17] as well as evidence for reduced lower extremity function one year after a hip fracture [12] provide additional impetus for ensuring that hip fracture patients are evaluated and optimally treated for vitamin D insufficiency.

Early in year 2, the Admission Initiative was amended to include administration of a single oral dose of 50,000 IU vitamin D_2_, the most available and inexpensive form of high-dose vitamin D. The rationale to initiate correction of vitamin D status as soon as possible was twofold: to ensure proper healing of the fracture and to address the underlying skeletal disease and risk of falls and subsequent fractures. Risk for fracture nonunion is also associated with metabolic and endocrine abnormalities. In a recent study in which adult patients with unexplained fracture nonunion were evaluated by an endocrinologist, the most common new diagnosis was vitamin D deficiency (68%), followed by thyroid abnormalities (24%), and hypogonadism (22%) [18]. In this study, history of hypothyroidism was noted in 13% of the hip fracture patients. Epidemiological data indicate the relationship between low vitamin D status and risk of falls and fracture [19]. A recent meta-analysis of the literature from randomized controlled trials revealed that trials that had used 700 or 800 IU per day of vitamin D plus calcium showed efficacy in reducing fracture risk by 26%, compared with calcium or placebo, whereas trials with 400 IU vitamin D/day were not effective [20]. This was reaffirmed in a larger meta-analysis that included data from 63 897 individuals [21]. Furthermore, from publications that included measurement of serum 25OHD, it is clear that antifracture efficacy increases with higher achieved 25(OH)D levels; levels above 30 ng/mL were achieved with 700–800 IU vitamin D_3_ [19].

There is some information about the co-occurrence of osteomalacia and osteoporosis in the elderly. Osteomalacia is diagnosed as the presence of excess osteoid and decreased mineralizing surfaces by histomorphometric analysis of nondecalcified histology. In a 1974 publication, histological osteomalacia in iliac crest biopsies was reported in 17% of women and 39% of men at the time of proximal femoral fracture [22]. In a 1990 histomorphometric analysis of iliac crest biopsies in elders with femoral neck fracture, osteoporosis was present in 71% and osteomalacia in 29% [23]. A recent histomorphometric study of biopsies from osteoporotic subjects showed an increase in osteoid thickness that was inversely correlated with serum 25OHD and emphasizes the importance of avoiding vitamin D insufficiency [24].

Although there are clinical tools to diagnose and therapeutic options to treat low bone density, osteoporosis remains underdiagnosed and seriously undertreated. Barriers to diagnosis and care of osteoporosis in patients with fragility fractures include uncertainties about clinical responsibility, the need for multidisciplinary medical teams, implementing computer-based tools, clinical expertise as recommendations for osteoporosis care evolve, and impact of other comorbidities upon treatment plan.

Various means have been suggested to enhance osteoporosis care in fracture patients, including interventions by primary care practitioners [25] and in outpatient postfracture clinical settings [26, 27]. In a recent report from a community-based multispecialty partnership, the delivery of osteoporosis care was increased after developing an information systems enhancement that automatically referred orthopedic fracture patients to an osteoporosis care service [28].

Less has been published about in-hospital initiatives, as they generally require paradigm shifts in large organizations. A retrospective analysis of hip fracture patients admitted to a tertiary care academic medical center in the United States showed that 17% were discharged on calcium in 2000, compared with 4% in 1995 [29]. In a series of patients hospitalized for fragility hip fracture in 2000, 10 of 75 (13%) were given a new prescription for osteoporosis medication while hospitalized [30]. A subsequent randomized study from that team compared effectiveness of two interventions during hospitalization: in the control intervention, patients received a pamphlet on fall prevention; in the study intervention, patients had a 15-minute discussion about osteoporosis evaluation with a research nurse and were provided with a list of questions for their primary medical physician about osteoporosis evaluation and treatment, followed with a telephone reminder 6 weeks after hospitalization about the need to address osteoporosis [31]. Evaluation by telephone at 6 months showed that 42% of those in the study group had received attention for osteoporosis, compared with 19% in the control group. Success was attributed to the impact of the research nurse's activities. Another osteoporosis consultation team reported that 53% of 59 patients with fragility hip fracture were not seen by the team because the initiating consultation request was not made [32]. Evaluation of three care models in different health-care environments also concluded that effectiveness increased when new personnel, such as an osteoporosis nurse practitioner, assumed the responsibility of coordinating patient education, laboratory tests, and communication between orthopedic staff and primary care physicians [33]. A pilot initiative from the American Orthopaedic Association used web-based training of orthopedic surgeons for process improvement measures and patient counseling, including calcium and vitamin D [34]. Those interventions had little effect on measures of osteoporosis management, but resulted in significant improvements in counseling and documenting the patients' osteoporosis risk factors. Expansion of that program was said to require new support mechanisms or personnel to ensure that presentation with an osteoporotic fracture launches appropriate standard of osteoporosis care [34].

This study highlights the importance of vitamin D in patients with fragility fractures. Identifying and correcting underlying osteoporosis in patients who present with fragility fracture are aimed to reducing the risk for additional fractures and disability [35, 36]. The consulting endocrinologist is the team member who is responsible for evaluating potential causes of secondary osteoporoses, in light of information that 80% or more hip fracture patients have medical conditions that are associated with increased fracture risk [36]. An in-hospital report of 25OHD ≤ 20 ng/mL is addressed with an additional 50,000 IU ergocalciferol before discharge and then 50,000 IU per week for another 6 weeks with retesting thereafter. An in-hospital level of 20 to 25 ng/mL is addressed with a prescription for 50,000 units twice per month. Higher doses of vitamin D repletion over shorter intervals are used by some physicians. The consulting endocrinologist also assesses risk for hypercalcemia, hypercalciuria, and nephrolithiasis with calcium and creatinine values and identifies patients with other contraindications for high-dose vitamin D, including sarcoidosis or granulomatous disease. Hypercalcemia is rare in the settings of hypovitaminosis D and low urinary calcium excretion, and, as we reported for an earlier series, patients with hip fracture tend to have lower circulating calcium levels than age and gender matched controls [8]. The endocrinologist also discusses with the patient the need for posthospitalization osteoporosis evaluation and management. An in-hospital prescription for weekly ergocalciferol is automatically carried forward into the discharge summary.

In this study, only 15% of subjects had a previous diagnosis of osteoporosis and some patients were admitted with a previous prescription for bisphosphonates. There are caveats to initiating antiresorptive osteoporosis medications during hospitalization for fragility fracture. The first concerns the evaluation and correction of vitamin D deficiency. Evidence indicates that bisphosphonates may produce hypocalcemia in the presence of vitamin D insufficiency [37] and that responsiveness to bisphosphonate is reduced in the presence of secondary hyperparathyroidism with vitamin D insufficiency [38]. Zoledronic acid (5 mg infusion once per year) is an option for patients with a recent hip fracture and is FDA-approved for secondary prevention of fractures. Recent evidence evaluating the timing of zoledronic acid administration post-hip fracture indicates decreased fracture reduction efficacy in subjects treated “within 2 weeks of their fracture repair,” compared with later treatment [39].

There is some information from animal studies about bisphosphonates not inhibiting early stages of fracture healing. Studies in ovariectomized rat models showed that administration of alendronate at the time of fracture resulted in larger callus formation, but with more woven than lamellar bone compared with control ovariectomized animals [40]. In a study with rats, a single dose of ^14^carbon-labeled zoledronic acid after a fracture demonstrated the greatest accumulation in the external fracture callus [41]. Although the clinical significance of those findings is not known, there is a concern that early intravenous administration of zoledronic acid would lead to greater uptake at the fracture site than in the rest of the skeleton and possibly interfere with remodeling and maturation of the healing bone.

In vitamin D sufficient hip fracture patients, in the absence of contraindications like esophagitis or gastric disorders, it may be practical to initiate therapy with oral bisphosphonates upon hospital discharge. It is not advisable to initiate annual intravenous bisphosphonate while in the hospital. For all bisphosphonates, vitamin D sufficiency should be documented before initiation of therapy; for intravenous zoledronic acid, it may be convenient to schedule the follow-up vitamin D test and the infusion at the time of outpatient appointments with the orthopedic surgeon or the endocrinologist.

In conclusion, effectiveness of in-hospital Admission and Discharge Initiatives requires communication and cooperation among orthopedic surgeons, endocrinologists, and other physicians, training and reinforcement, computer assistance, audits, and evolution. This study shows the beneficial results of self-evaluation and systems analyses to identify opportunities for improvement. The prevalence of vitamin D insufficiency observed in this study reaffirms the importance of incorporating vitamin D recommendations in fragility fracture care initiatives. Orthopedic surgeons who treat patients with acute fragility fracture are in ideal position to initiate in-hospital multidisciplinary care pathways aimed at reducing the sequelae and preventing other fractures.

## Figures and Tables

**Table 1:** Comparison of effectiveness of B-FIT admission and discharge initiatives in subjects admitted with hip fragility fracture. (i) Admission initiative was introduced early in Year 1; (ii) admission initiative was modified early in Year 2 with a computer-assisted reminder; (iii) discharge initiative was introduced one year before Year 1 with computer-based directives.

	Year 1 cohort	Year 2 cohort	Year 3 cohort	Year 4 cohort
Number of subjects	57	41	42	41

Age (years, mean ± SD, median)	77.5 ± 11.0	73.9 ± 12.8	79.2 ± 10.7	79.1 ± 11.8
	81	74	82	81

Percent, number women	74%, 42	71%, 29	76%, 32	78%, 32

Effectiveness of admission initiative	33% (19/57)	41% (17/41)	57% (24/42)	68% (28/41)

Analysis of effectiveness of admission initiative; *P*-*values *		*.524 versus Year 1*	* .024 versus Year 1*	* .001 versus Year 1 *
			*.190 versus Year 2*	*.026 versus Year 2*
			*.040 versus Years 1 and 2*	*.366 versus Year 3*
				*.001 versus Years 1 and 2*

Effectiveness of discharge initiative	67% (38/57)	82% (32/39)	83% (35/42)	80% (32/40)

Analysis of effectiveness of discharge initiative; *P* *-values *		* .108* *versus* *Year 1*	*.07 versus Year 1*	*.17 versus Year 1*
			*1.00 versus Year 2*	*1.00 versus Year 2*
			*1.00 versus Years 1 and 2*	*.78 versus Year 3*
				*.52 versus Years 1 and 2*
